# Case study of a landslide continuous probability rainfall threshold analysis based on the prediction interval principle

**DOI:** 10.1038/s41598-023-29625-6

**Published:** 2023-02-10

**Authors:** Yu Huang, Cuizhu Zhao, Xiaoyan Jin, Yan Zhu, Ming Peng, Zhiyi Chen

**Affiliations:** 1 Department of Geotechnical Engineering, College of Civil Engineering, Tongji University, Shanghai, 200092 China; 2 Key Laboratory of Geotechnical and Underground Engineering of the Ministry of Education, Tongji University, Shanghai, 200092 China; 3 Shanghai Research Center of Ocean & Shipbuilding Engineering, China Shipbuilding NDRI Engineering Co., Ltd., Shanghai, 200090 China

**Keywords:** Natural hazards, Civil engineering

## Abstract

Bazhong City is located on stratum dominated by red-bed conditions. This type of weak geological condition with sand and mudstone interbedding is very easily affected by climatic conditions to produce rainfall-type landslides. On the basis of landslide data statistics collected in Bazhong City from 2011 to 2019, this paper uses ERA5-Land rainfall data to statistically analyze the correlation between rainfall and landslide events in Bazhong City. The landslide events in Bazhong City are greatly affected by rainfall events lasting for 10 days. Considering the influence of rainfall seepage and other processes, an effective cumulative rainfall-duration threshold curve for Bazhong City is obtained via median nonlinear fitting. Then, on the basis of the prediction interval, the rainfall threshold formula under different landslide occurrence probabilities is obtained and the critical threshold curve with a non-exceeding probability of 1% in Bazhong City is calculated and verified. Subsequently, a continuous probability distribution fitting function of landslide occurrence is established and a continuous probability distribution surface with a good fitting effect in Bazhong City is obtained. This allows a definite probability of whether future rainfall events will induce landslides to be obtained, providing an important basis for engineering disaster prevention and mitigation.

## Introduction

Landslides are among the most widely distributed, most influential, and most damaging geological disasters worldwide, and rainfall is one of the most important factors inducing landslides^1^. Rainfall not only increases the weight of the landslide mass but also reduces the shear strength of the slope, reducing the slope stability^2^. Currently, studies concerning the impact of rainfall on the landslide stability primarily focus on two aspects: conducting stability calculations or model tests on a certain slope^3–5^ and establishing landslide rainfall thresholds based on regional historical landslide rainfall data statistics^6–8^.

Especially in mountainous areas, the number of slopes is large, the range is large, and data are lacking. Therefore, whether a rainfall event will induce a landslide is difficult to judge. Because of the large number of potentially unstable slopes, performing data measurements and monitoring for every slope are extremely difficult, resulting in a lack of analysis data. Consequently, an appropriate assessment method needs to be selected. Rainfall threshold research based on regional historical occurrences can provide a basis for landslide warnings. The most basic requirement is to have many years of landslide and rainfall data and then to analyze the statistical relationship between the occurrence of landslide events and the rainfall conditions to obtain the rainfall threshold that induces a landslide event^9^. The rainfall threshold is a critical rainfall parameter value that characterizes an induced landslide. This concept was first proposed by Caine^10^, who compiled 73 cases of rainfall-induced landslides and debris flows, characterized by the rainfall duration and the average rainfall intensity, and used the envelope under a scatterplot of the rainfall-induced landslides as the rainfall threshold. This is the now widely used rainfall intensity–duration threshold concept^10,11^. Of course, the current concept of rainfall thresholds also includes the cumulative rainfall–duration threshold^12–14^, cumulative rainfall–rainfall intensity threshold^15^, and total rainfall threshold based on rainfall-induced landslides.

A rainfall-induced landslide is a dynamic process that includes the gradual infiltration of rainfall and the gradual saturation of the rock and soil. In the case of a low rainfall amount with a long duration, the process of inducing landslides has a strong time series and a certain hysteresis. The impact of rainfall on landslide hazards includes two factors: rainfall duration and previous rainfall amount. Therefore, in a rainfall threshold analysis, the previous rainfall duration should first be determined. Studies have been conducted concerning the selection of the parameters for the previous rainfall duration, but there is no consensus at present. The length of the previous effective rainfall duration, as determined by different authors, varies greatly. For example, Wieczorek et al. determined a time of rainfall-induced slope instability of 5 days^16^, while Kim et al. established the impact of early rainfall as being up to 20 days^17^. The early rainfall is greatly affected by the selected range of the rainfall duration study; therefore, the length of the rainfall duration should be defined first in a threshold analysis.

The occurrence of rainfall-induced landslides has great uncertainty. A traditional threshold analysis can only roughly estimate whether a landslide can be induced in a certain region under certain rainfall conditions based on experience. Therefore, in the process of threshold calculation and analysis, the 5% non-exceeding probability rainfall threshold is usually selected^7,18–20^. However, miscalculations may be associated with this threshold. In current research, some studies have given the upper and lower limits of the rainfall threshold^21^, while others have calculated the rainfall thresholds of different probability levels^22–24^. The lower threshold refers to the record lower than that without a rainfall-induced landslide, which means that the slope will not fail until a certain rainfall intensity or rainfall amount is reached. The upper threshold refers to the limit of rainfall-induced landslide events, which both depends on extreme meteorological disasters and includes the failure of strongly stable slopes. Between these thresholds, there are different landslide occurrence probabilities that need to be quantified.

Bazhong City is in the northeastern part of the Sichuan Basin, where mountains and hills are widely distributed, accounting for 98.2% of the total area. The terrain in the region varies greatly, the stratum and lithology are complex, and there are developed fold structures. The climate in this region is complex and changeable, and the geological disasters are multi-faceted, with strong concealment, significant danger, and considerable potential damage. According to statistics from the Bazhong Municipal Bureau of Land and Resources, by the end of 2014, there were 4432 hidden hazard points that could threaten the lives and property of residents in Bazhong City. These identified geological hazards primarily include landslides, debris flows, collapses, and potentially unstable slopes. Landslide geological disasters constituted the majority. According to statistics, there are 4014 landslide hazards, accounting for 90.6% of the total^25^. The density of hidden hazards in Bazhong City ranks second in Sichuan Province, and the number of people threatened per unit area ranks first in the province, with a maximum of 16.5 people threatened per square kilometer, which is approximately four times the average in Sichuan Province. This means that the lives of more than 240,000 people and property valued at nearly 5.4 billion yuan are threatened to varying degrees. One of the reasons for these hidden hazards is that the lithology of Bazhong City consists of primarily Triassic–Jurassic sand–mudstone interbeds^26^. This is a typical soft and hard red-bed rock mass, having characteristics of low strength, easy weathering, poor hydraulic properties, and large deformation. At the same time, the water resistance of mudstone and shale causes small particles carried by infiltrating rainwater to slowly discharge along the interface with the sand layer. Under the action of dry and wet cycles, weak siltized intercalation is formed, which can easily develop into an interlayer shear zone, causing slope instability. As a result of this special geological condition, large numbers of landslides occur in Bazhong City under rainfall conditions. This has a significant impact on the lives of residents. According to existing research^26^, landslide hazards in Bazhong City are primarily small and shallow soil landslides, which are highly sensitive to rainfall. Landslide areas are generally in a basically stable state under natural conditions and are prone to slip deformation under heavy rainfall conditions. Landslides in this region have small sizes, occur in large quantities, have high density, are prone to failure, and have an uneven spatial and temporal distribution.

The “September 16 torrential rain event” in 2011 triggered a large number landslides in Bazhong City, Nanjiang County, reaching a total of 1162 slides; this event inspired multiple studies concerning the rainfall threshold in red-bed areas. Taking the Erhuangping landslide in Nanjiang County as an example, Lu et al. analyzed the impact of rainfall on soil landslides in a red-bed area and obtained a fitting formula for the relationship between the rainfall intensity and the duration^27^. At the same time, in the process of establishing a prediction model for large-displacement landslides induced by heavy rainfall, Yu et al. produced two rainfall threshold curves for Nanjiang County^28^. In addition, on the basis of the rainfall duration and rainfall process, Zhang et al. discussed the rainfall types that trigger landslides in Bazhong City and found that such rainfall types were primarily incremental and unimodal^29^. However, there is no specific research concerning the rainfall threshold conditions for landslide occurrences in Bazhong City.

From June 19 to 20, 2019, Bazhong City was again hit by a heavy rainfall event. The maximum rainfall was 196.1 mm (from 20:00 on June 19 to 7:00 on June 20) in Guangong Town, Enyang District. The heavy rainfall caused floods in Bazhong City and greatly elevated the risk of landslides on the local mountains and slopes. Following the heavy rainfall, a large number of statistical investigations concerning landslide incidents in Bazhong City were performed. A total of 165 slope failures were identified from June to July. This event was sufficient to draw attention to rainfall-induced landslide events in Bazhong City. Because of differences in the geotechnical properties, slope gradients, and deterioration degrees of slopes, there will always be differences in whether and when landslides are induced on a slope given the same type of rainfall event. Therefore, it is necessary to perform threshold analyses using probability levels and statistics. In particular, obtaining the threshold curve under a certain non-exceeding probability and the landslide probability of different rainfall events is of great significance to provide early warnings of slope engineering disasters in this region.

Here, we study the rainfall threshold that triggers landslides in the area of Bazhong City, Sichuan Province, China, which is prone to rainfall-type landslides. By statistically analyzing the collected rainfall and landslide data from 2011 to 2019, a calculation formula of the threshold curve under different probabilities can be obtained. We then calculate and verify the threshold under a non-exceeding probability of 1%. Accordingly, a fitting formula for the continuous probability distribution of the landslide events is established. Using the effective cumulative rainfall and the previous rainfall duration, the probability of landslide events can be evaluated over time, providing a basis for forecasts and early warnings of slope geological disasters in Bazhong City.

## Study area and materials

### Study area

**Figure 1:**
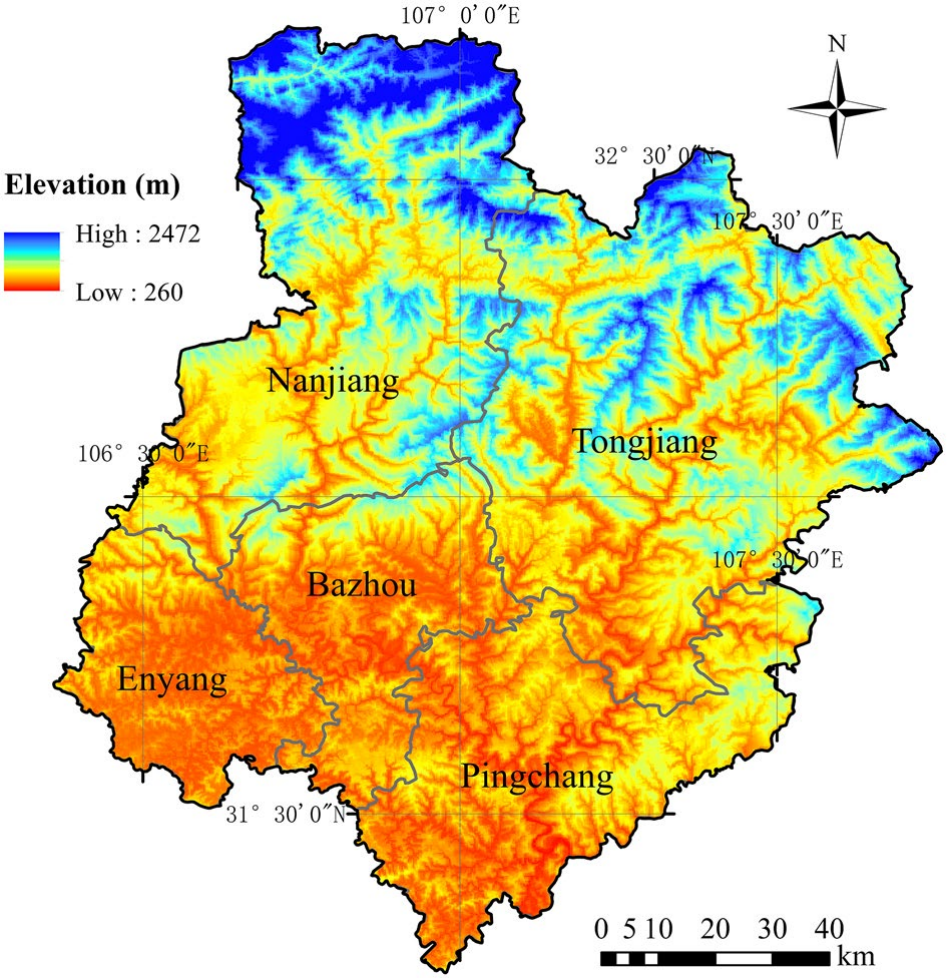
Elevation Map of Bazhong City, Sichuan Province, China.

Bazhong City is located in the northeastern part of Sichuan Province, south of the boundary between the Qinling Mountains and the Huaihe River in China, bordering Dazhou in the east, Nanchong in the south, Guangyuan in the west, and Hanzhong in Shaanxi in the north. Its specific geographical location is shown in Fig. 1, between 106° 20′ E and 107° 49′ E longitude and between 31° 15′ N and 32° 45′ N latitude, with an area of 12,300 km^2^. Bazhong City has a total of five county-level administrative divisions, including two municipal districts and three counties.

**Figure 2:**
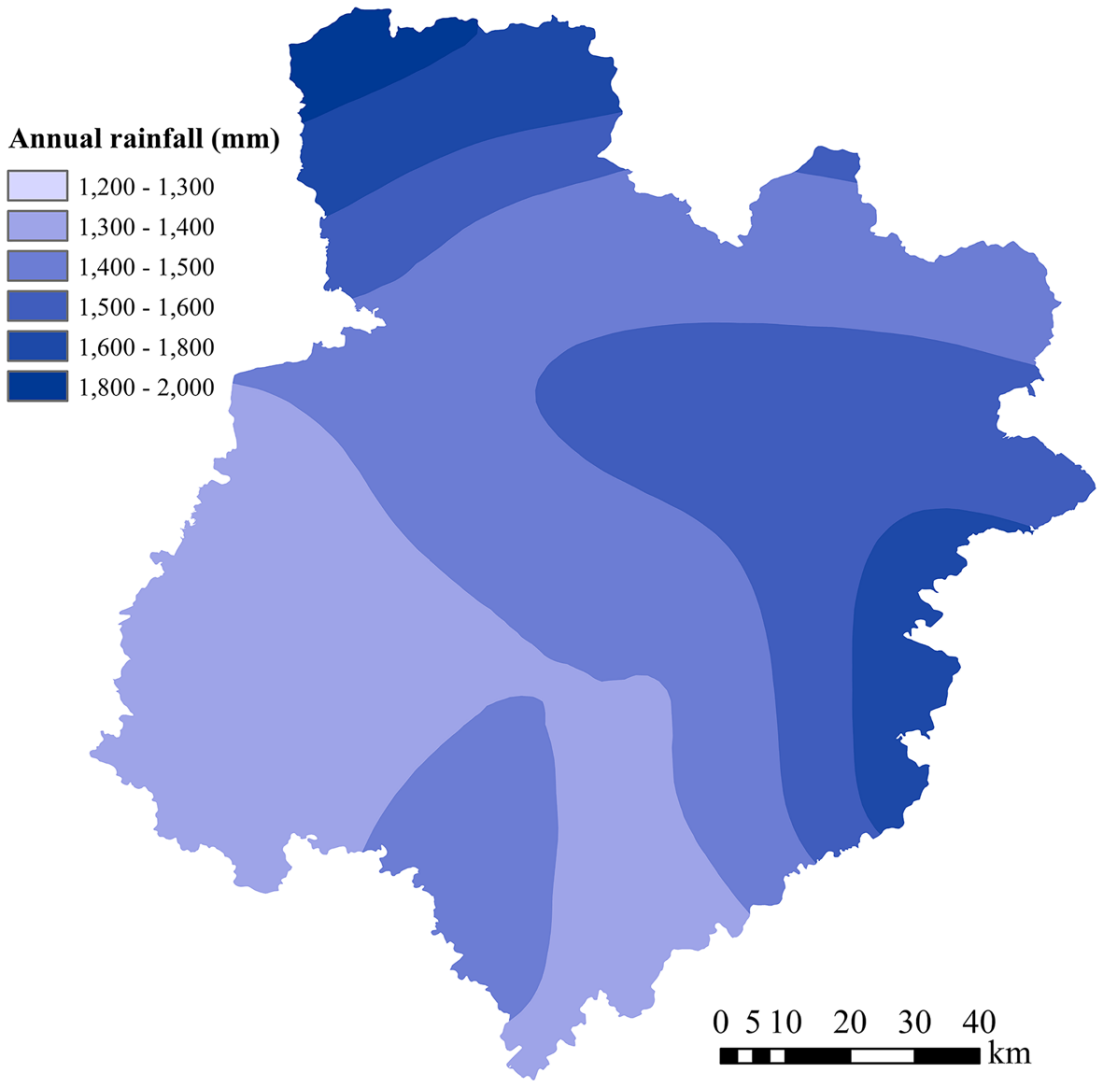
Average annual rainfall distribution from 2011 to 2019.

Bazhong City is located in a typical mountainous area, with high terrain in the north and low terrain in the south, as shown in Fig. 1. The terrain has a minimum elevation of 268.3 m in Huangmeixi, Pingchang County, in the south. The highest altitude is 2507 m in Guangwu Mountain, Nanjiang County, in the northwest. There are many mountains and hills in the study area; therefore, the area includes flowing water erosion, sedimentation, fan-shaped landform and gravity accumulation, residual landforms, and karst landforms. Bazhong City belongs to a typical subtropical humid monsoon climate zone, with abundant rainfall and a mild climate. According to the Bazhong City Yearbook, the average precipitation from 2011 to 2019 was 1229 mm. The annual rainfall in Nanjiang County was the largest in 2011 at 1910.1 mm, and the annual rainfall in Enyang District in 2015 was the smallest at 838.5 mm. The average annual rainfall distribution calculated based on the rainfall data from 2011 to 2019 is shown in Fig. 2, which shows a decrease from southeast to northwest resulting from the combined influence of the monsoon climate and the topography.

### Data

**Figure 3:**
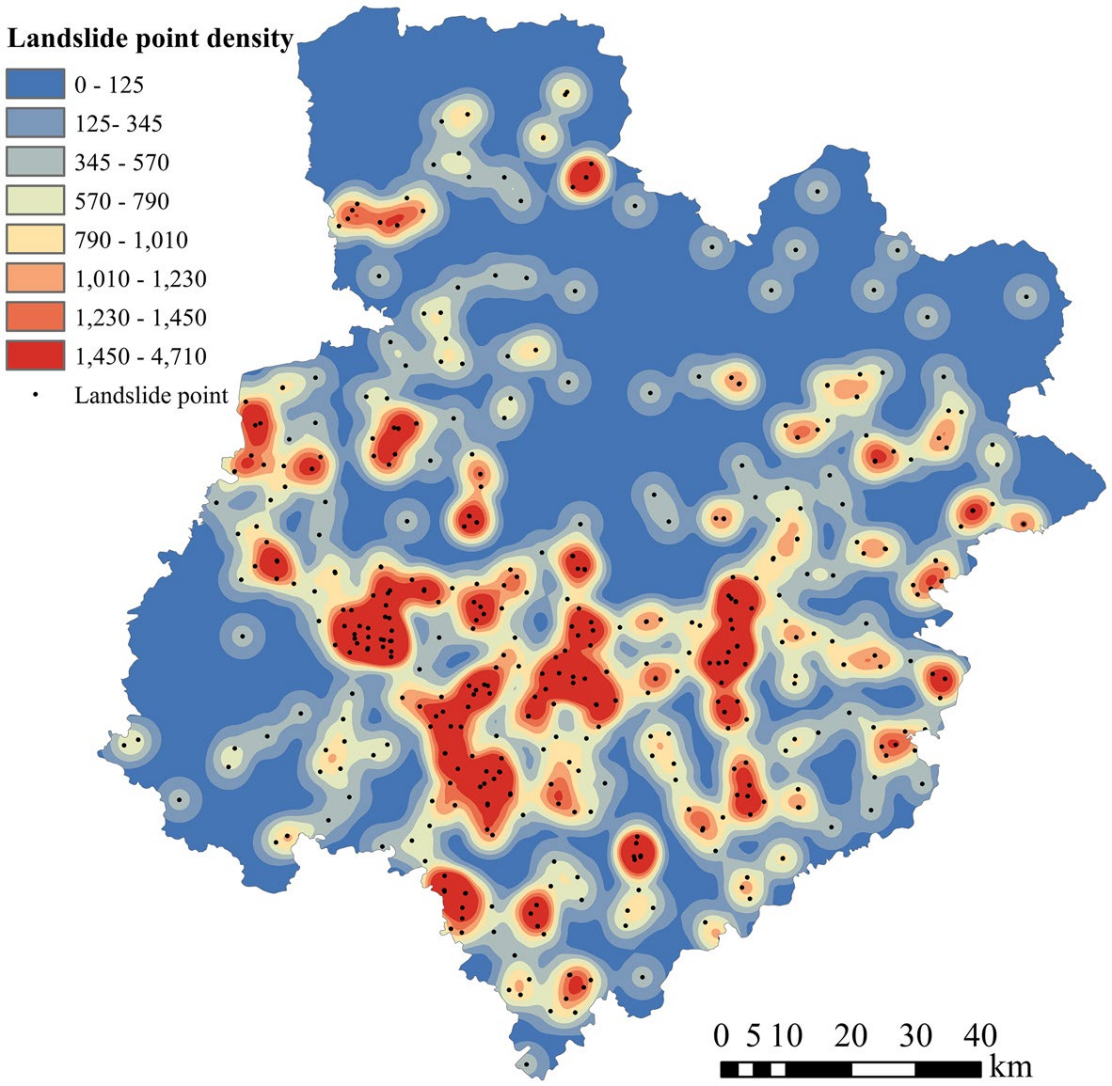
Distribution map of landslide hazard points in Bazhong City from 2011 to 2019.

This study collected landslide hazard data in Bazhong City from 2011 to 2019. Part of the data comes from the Natural Resources and Planning Bureau of Bazhong City, Sichuan Province, and another part comes from the collection of thesis materials^30^. A total of 515 landslide hazard events were collected in five counties and districts over 9 years; the distribution of these events is shown in Fig. 3. In this study, only the approximate point of landslide occurrence is obtained, regardless of its influence range. According to the landslide data statistics, we primarily considered several situations, such as slope instabilities caused by rainfall, induced landslides or debris flows, and further aggravated landslides. In addition, these landslide events pose potential harm to human engineering activities, or have caused harm, including catastrophic landslide events and slope instability events that have not yet developed into disasters. The point density in Fig. 3 is calculated using the kernel density estimation method^31^. The data in the legend represent the number of landslide points per square kilometer.

The acquisition of rainfall data includes ground station observations, radar observations, and satellite remote-sensing observations. Ground-observed rainfall data are limited by the number of stations established. Radar-estimated precipitation is affected by radar wave attenuation and complex terrain occlusion, and the obtained temporal and spatial coverage is limited. The ERA5 land precipitation data used in this paper have a resolution of 0.1° × 0.1°. A physical model based on global observations was used for the reanalysis. These climate data have high accuracy. The ability of the dataset to capture precipitation events is good; this has certain advantages for the daily-scale analysis required in this study and, most importantly, makes it easy to obtain data^32,33^. However, no matter how high the precision of the raster data, values can only be provided on a certain latitude and longitude grid^34,35^. In the process of extracting rainfall data corresponding to landslide events at specific spatial locations and time points, spatial linear interpolation was further adopted, ignoring the non-stationary variability of the rainfall in the spatial and temporal distribution^36^.

### Statistical analysis

**Figure 4:**
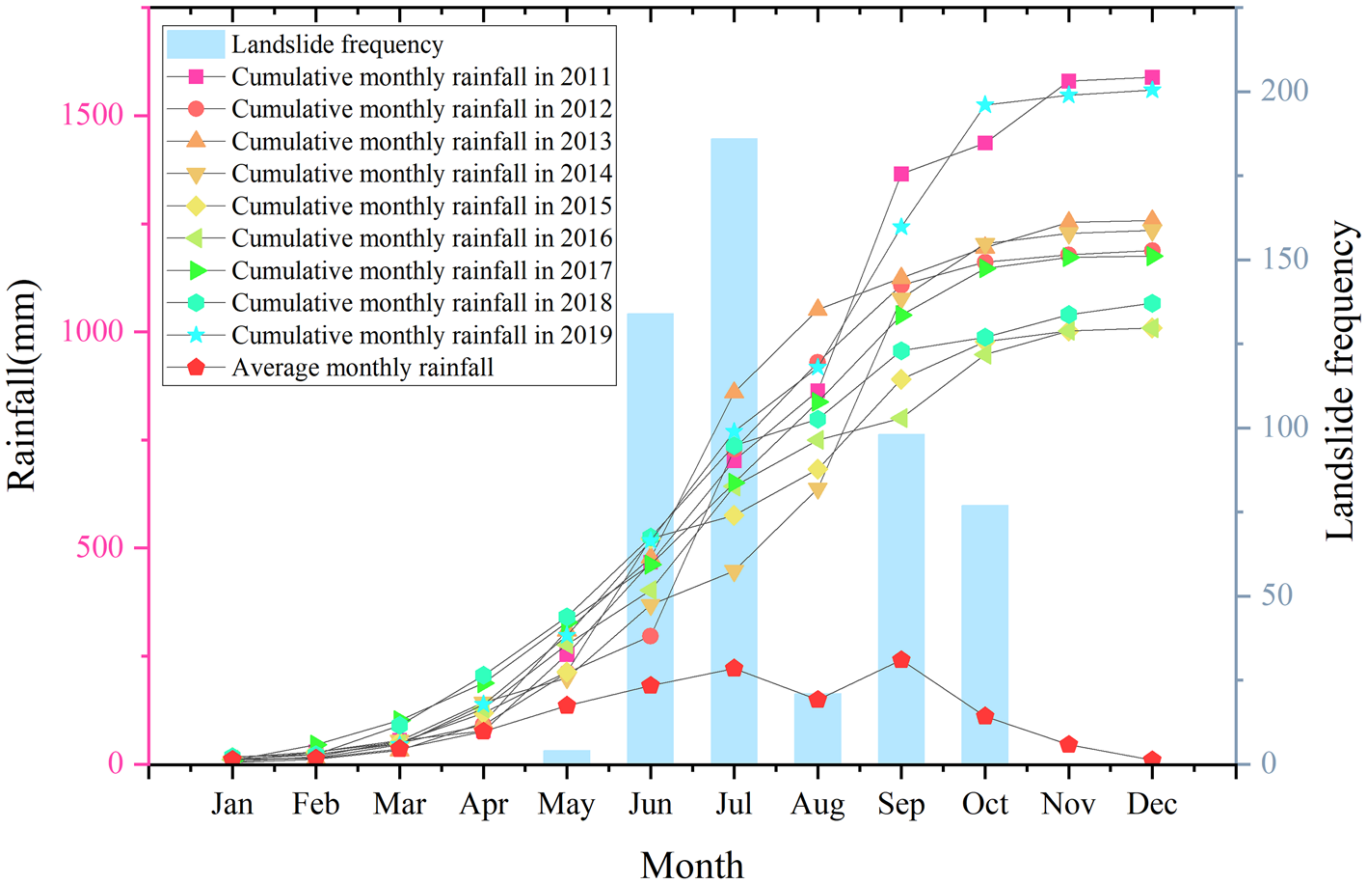
Monthly rainfall and landslide hazard frequency from 2011 to 2019.

According to the Bazhong City Yearbook, statistics were compiled for the average monthly rainfall and the occurrence time of landslide hazards over many years, as shown in Fig. 4. Landslide hazards primarily occur from May to October, with the number of disasters generally increasing with increasing rainfall and decreasing with decreasing rainfall, indicating that there is a close relationship between the two.

To illustrate the relationship between landslide hazards and daily rainfall, this study performed frequency statistics of landslide hazards according to the rainfall grades shown in Table 1^37^. In addition, even though a total of 515 landslide hazards were identified in Bazhong City from 2011 to 2019, many landslide events occurred on the same day. To better explain the relationship between landslide hazards and daily rainfall, this study de-duplicated the number of landslides that occurred on the same day; that is, all landslide events that occurred during one day were recorded as a single disaster day.

**Table 1:** Rainfall intensity distribution statistics of landslide disaster days.

Level	Daily rainfall (mm/day)	Number of landslide days
No rain	0	11
Light rain	(0, 10)	43
Moderate rain	[10, 25)	20
Heavy rain	[25, 50)	11
Rainstorm	[50, 150)	16

Judging from the number of landslide days, it can be seen that, when the daily rainfall exceeds 50 mm, the number of disaster days increases. The data indicate that landslide hazards are more likely to occur with heavy rainfall weather. However, the collected landslide events indicate that the frequency of landslide hazards is approximately 50% when there is no rain or light rain on the disaster day. There are two reasons why the analysis produces this result. The first reason is that the collected landslide events are not induced by rainfall factors or that they are potential landslide events that have been in a state of creep. It is necessary to screen landslide events on the basis of a comprehensive analysis of rainfall events and landslides to obtain the rainfall-type landslide events. The second reason is that the accumulation of precipitation and seepage has a significant influence on the induced landslide events. This reason is very important; the concepts of rainfall intensity duration threshold, cumulative rainfall intensity threshold, and total rainfall threshold emphasize the impact of slope instabilities caused by early rainfall accumulation.

**Figure 5:**
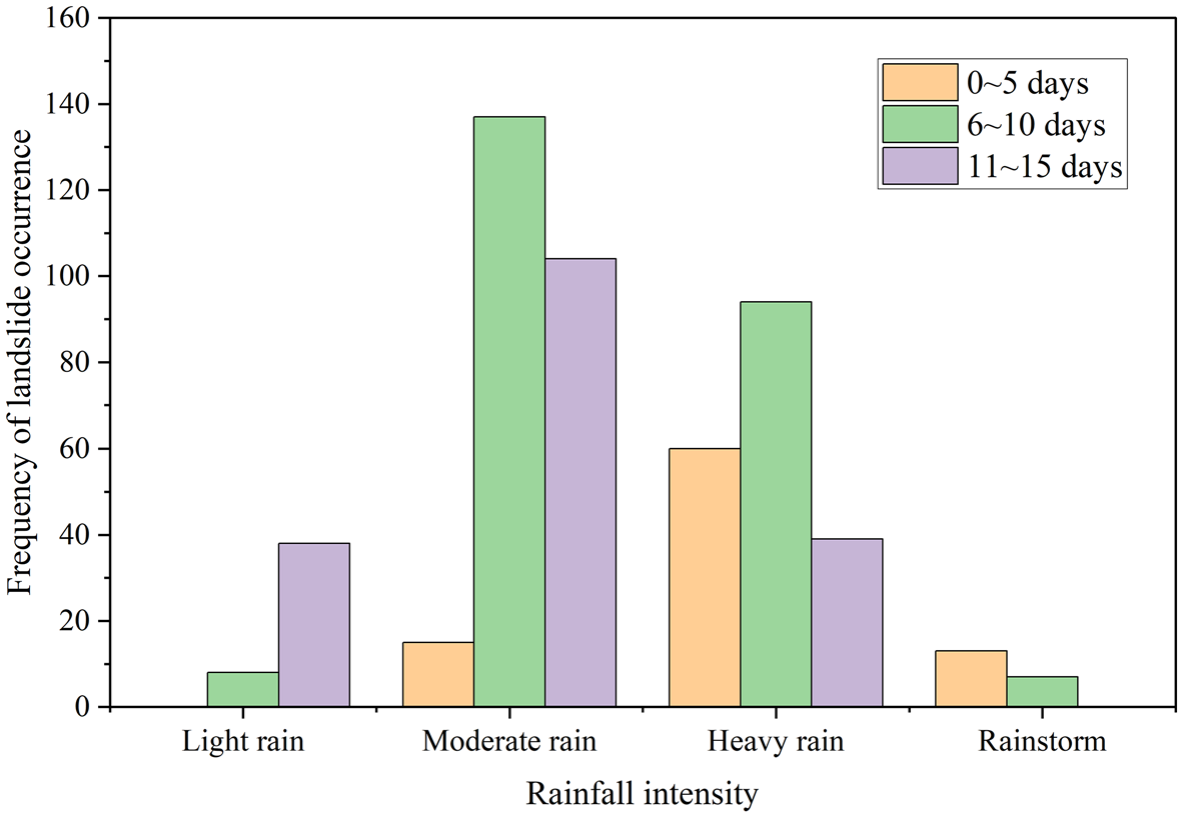
Relationship between the average daily rainfall in the previous period and the frequency of landslides.

Statistics were acquired with respect to the grades of the average daily rainfall in the previous stage of the induced landslides, as shown in Fig. 5. It can be seen that, when the previous rainfall lasted for 5 days, the daily average rainfall was primarily heavy rain. However, the rainfall events that triggered landslides in Bazhong City were primarily moderate rainfall events lasting 6–10 days. It can be seen from this that the landslides in Bazhong City have a strong relationship with the early accumulated rainfall. When calculating the rainfall threshold, at least 10 days of rainfall duration are taken for the analysis. Therefore, in terms of the subsequent selection of the rainfall threshold, we counted and analyzed the rainfall data 15 days prior to the landslide event. This includes most landslide events induced by continuous rainfall and reduces the statistical workload of the rainfall data to a certain extent.

## Methods

To obtain the probability of a slope instability landslide under a certain rainfall condition, we propose a further fitting method to obtain the continuous probability threshold surface based on existing rainfall threshold research. The main process is as follows.

1. Using the previous data statistics and analysis, select the appropriate rainfall threshold model. In this study, the importance of early rainfall is evident, so the cumulative rainfall duration threshold model is adopted.
2. Perform a power law function nonlinear fitting of the rainfall threshold. At the same time, calculate the standard deviation of the residual difference and statistically analyze its distribution.
3. On the basis of the prediction interval principle, the rainfall threshold under any non-exceedance probability is obtained through the inverse operation of the cumulative distribution function. For residuals that do not conform to normal distribution, the normal distribution should be converted.
4. By fitting the threshold surface, the calculation formula of the landslide occurrence probability under arbitrary rainfall can be obtained.

### Rainfall threshold

The frequency of landslide hazards does not always increase with increasing rainfall duration. That is, in a long-term rainfall process, not all rainfall impacts the occurrence of landslides. In the process of rainfall, there are effects such as runoff, discharge, and evaporation, especially with increasing duration, and these effects cannot be ignored. To overcome this problem, Crozier proposed using the effective cumulative rainfall (${E}_{\mathrm{L}}$) to represent the impact of cumulative rainfall events; this method is widely used^38,39^:

1 $${E}_{\mathrm{L}}={E}_{\mathrm{L}}(0)+k{E}_{\mathrm{L}}(1)+{k}^{2}{E}_{\mathrm{L}}(2)+\dots +{k}^{N}{E}_{\mathrm{L}}(N)={\sum }_{i=0}^{N} {k}^{i}\left[{E}_{\mathrm{L}}(i)\right].$$

In Eq. (1), ${E}_{\mathrm{L}}(0)$ is the rainfall on the day of the landslide; ${E}_{\mathrm{L}}(i)$ is the daily rainfall on the *i*th day prior to the landslide; $N$ is the number of days of previous rainfall; and $k$ is the effective rainfall coefficient, whose value ranges from 0.6 to 0.93^40,41^, depending primarily on the local rainfall permeability. In most studies, a value of *k* of 0.8 is used^17,42–44^. Because the occurrence of landslides in Bazhong City is primarily affected by rainfall events within 10 days and the influence of rainfall prior to 11–15 days is ignored, the *k* value in this study was taken to be 0.84^38,45,46^. At this value, 17% of the precipitation on the 10th day contributes to the occurrence of landslides.

On the basis of the formula of the effective cumulative rainfall, the relationship between the effective cumulative rainfall and the rainfall duration was derived^14^; that is, the model shown in Eq. (2) is the rainfall threshold in Bazhong City. The rainfall duration was taken as the period from the first day of the previous rainfall duration to the day when the landslide occurred; the analysis was performed in units of days.

2 $${E}_{\mathrm{L}}=\left(\mathrm{\alpha }\pm \Delta \mathrm{\alpha }\right)\times {D}^{\left(\upgamma \pm \Delta\upgamma \right)}.$$

In Eq. (2), $\mathrm{\alpha }$ is the scaling constant, which is the intercept of the threshold curve; $\upgamma$ is the shape parameter, which defines the slope of the power function curve; and $\Delta \mathrm{\alpha }$ and $\Delta\upgamma$ represent the uncertainties in $\mathrm{\alpha }$ and $\upgamma$, respectively^47^. This equation can be directly obtained via nonlinear fitting and can provide the fitting parameter range under a certain confidence level.

### Prediction interval principle

In previous analyses, to obtain the landslide threshold under a certain non-exceeding probability, the frequentist method has usually been used^48^. Similarly, the principle of the prediction interval can provide predictions according to certain methods and rules based on the collected rainfall-induced landslide events, so as to determine the occurrence or probability of landslides in advance. Assuming that the prediction errors are normally distributed, the prediction interval at a confidence level of $1-2\alpha$ is

3 $$\left[\mathrm{lower},\mathrm{upper}\right]=\left[\widehat{y}+{Z}_{\alpha }\sigma ,\widehat{y}+{Z}_{1-\alpha }\sigma \right].$$

In Eq. (3), $\widehat{y}$ represents the fitted value for a given independent variable *x* in the regression analysis; $\sigma$ is the standard deviation of the residual obtained in the process of fitting the power function; and $Z$ represents the distance from the mean to zero with a probability of $\alpha$ or 1 − $\alpha$ in the standard normal distribution. According to the empirical value of a normal distribution, when $\alpha =5\mathrm{\%}$, $\mathrm{Z}=-1.64$ and, when $\alpha =95\mathrm{\%}$, $\mathrm{Z}=1.64$.

To obtain the threshold curve under a certain non-exceeding probability in a landslide-induced rainfall threshold analysis, it is first necessary to obtain the $Z$ value. This can be obtained via the inverse cumulative distribution function of a normal distribution, which is ${Z}_{\alpha }={\Phi }^{-1}(\alpha )$. The cumulative distribution function of a normal distribution is

4 $$\Phi \left(z\right)=\int_{-\infty}^{z}\frac{1}{\sqrt{2\pi {\sigma }^{2}}}{e}^{-\frac{(x-\mu )}{2{\sigma }^{2}}}dx.$$

In Eq. (4), $\mu$ is the mean value of the residuals in the regression fit. The inverse function of this equation is defined in many programs, for example, it can be obtained via the “norminv” function in the MATLAB software.

In addition, for a function whose fitting residuals do not conform to a normal distribution, function conversion is required. For the fitting of the rainfall threshold, the power law function is generally used and the residuals generally conform to a lognormal distribution. Accordingly, Eq. (3), based on the prediction interval principle, was modified to obtain a non-exceeding probability calculation formula that conforms to the rainfall threshold analysis:

5 $${E}_{L-\alpha }=a\cdot {Z}_{\alpha }{\sigma }_{lgR}\cdot {D}^{b}.$$

In Eq. (5), ${\sigma }_{lgR}$ is the standard deviation of the logarithmic residual. The ${Z}_{\alpha }{\sigma }_{lgR}$ component can be directly expressed as the inverse function ${\Phi }^{-1}(\alpha )$ of a normal distribution, and the normal distribution $\Phi \left(z\right)$ obeys the logarithmic residual mean and standard deviation distribution. In this context, in the rainfall threshold analysis, the mean and standard deviation obeyed by the logarithmic residual need to be obtained first via a nonlinear fitting of the median. Then, the threshold curve under any non-exceeding probability can be obtained using Eq. (5).

### Continuous probability distribution model

To more accurately and conveniently prevent future rainfall-type landslide events in Bazhong City, it is necessary to analyze the continuous probability of landslide events in this region. Because the probability distribution function is a sigmoid function, this study establishes the following binary function to characterize the effect of the effective rainfall accumulation and duration on the probability of landslide occurrence:

6 $$P=\frac{1}{1+{e}^{a+bx+cy}}.$$

In this formula, $x$ and $y$ are independent variables representing the effective accumulation of rainfall and the rainfall duration, respectively; and $a$, $b$, and $c$ are fitting parameters.

## Results and validation

According to the analysis results of the aforementioned landslide hazards and rainfall data, landslide hazards in Bazhong City are dominated by moderate rain lasting approximately 10 days. The landslide rainfall data were rectified considering the influence of non-rainfall-induced landslides. Data for landslides induced by 15 days of continuous rainfall in the early stage were not included because it is unclear whether the rainfall lasted for 15 days or longer; that is, this part of the data was not sufficiently complete, which has a certain impact on the analysis results. Considering the effective rainfall and the previous rainfall duration, a total of 391 landslide rainfall disaster records were screened. In this study, rainfall-induced landslide events were divided into two datasets. One portion of the data was used for threshold curve fitting, and the other portion was used to validate the threshold results. This study used landslide data from January 2011 to August 2019, with a total of 329 records, to calculate the rainfall threshold. At the same time, to calibrate the threshold, we counted 775 non-landslide points during the period from January 2011 to June 2019 when no landslides were induced.

### Landslide rainfall threshold

**Figure 6:**
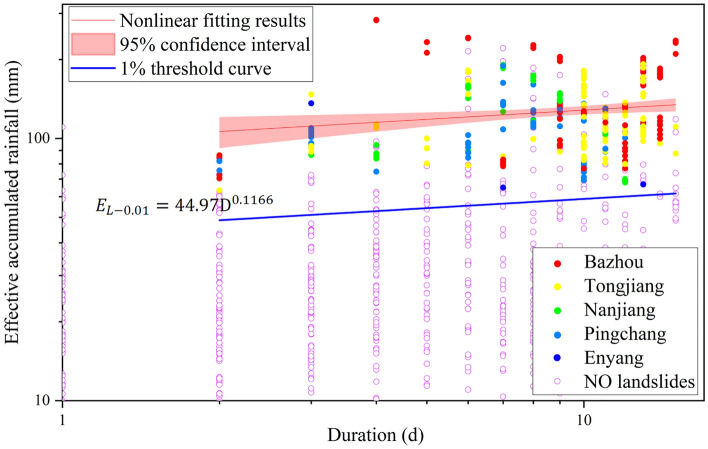
Landslide rainfall threshold curve for Bazhong City.

Median nonlinear fitting based on a power function was performed on the 329 screened records, and the results are shown in Fig. 6. The distribution of rainfall-type landslide events in Bazhong City is relatively discrete, and the fitting effect is not very good. Therefore, considering the uncertainty of the fitting, the fitting confidence interval with a confidence level of 95% and the confidence ranges of the coefficients $\mathrm{\alpha }$ and $\upgamma$ were calculated. The confidence intervals of the fitted results are shown in the shaded region in Fig. 6. Considering the uncertainty of the fitting coefficients $\mathrm{\alpha }$ and $\upgamma$, the median nonlinear fitting threshold curve and the standard deviation of the fitting parameters are expressed as

7 $${E}_{\mathrm{L}}=\left(98.05\pm 9.68\right){\mathrm{D}}^{0.1166\pm 0.0442}.$$

Figure 6 shows that the use of a 1% non-exceeding probability threshold has a poor early warning effect, with 107 landslide rainfall events not triggered. However, compared with the rainfall threshold fitted by the median line (50% non-exceeding probability threshold), it has a good early warning effect and only 18 heavy rainfall events did not induce landslides. This comparison indicates that the rainfall threshold with nonlinear fitting has a better warning effect.

**Figure 7:**
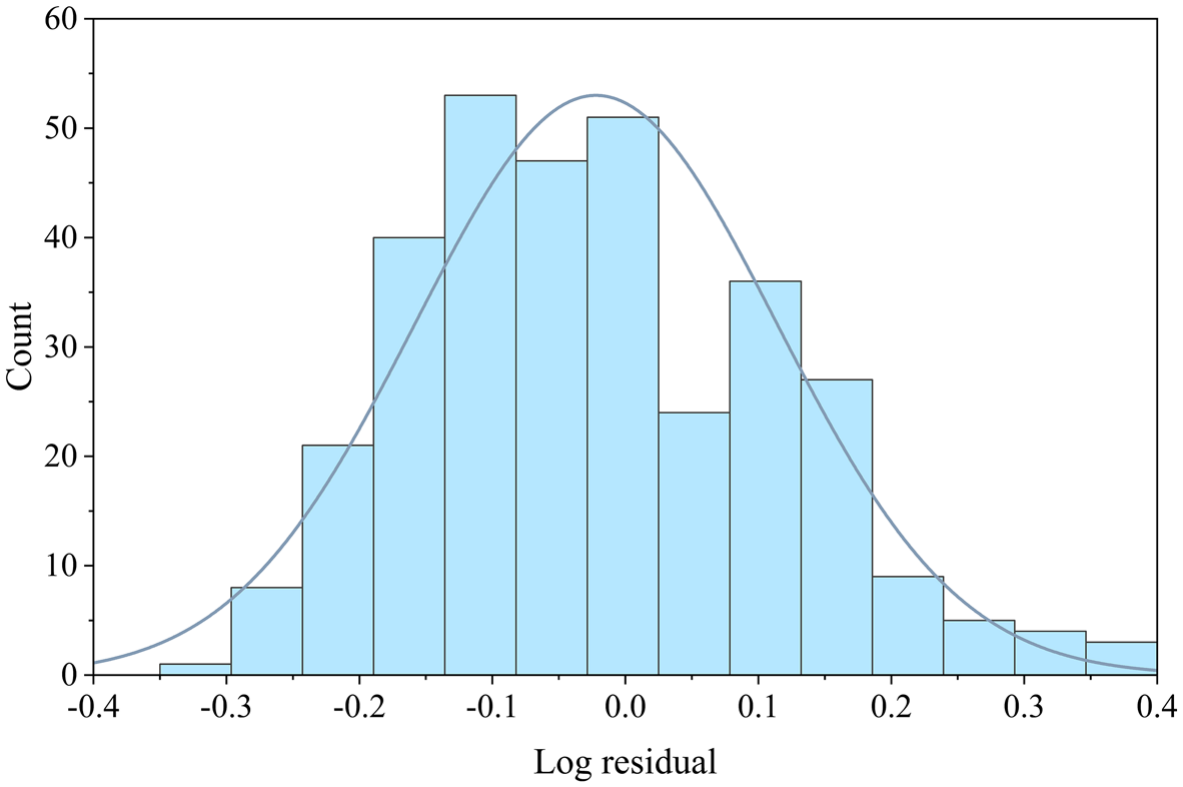
Logarithmic residual distribution of the rainfall threshold curve fitting for Bazhong City.

To illustrate the validity of the rainfall threshold curve obtained by the nonlinear regression analysis, a residual analysis was performed on the actual effective cumulative rainfall value and the value obtained via fitting. Because this study uses a power function relationship to characterize the rainfall duration and the effective cumulative rainfall that induces landslides, the frequency distribution of the logarithmic residuals was calculated, as shown in Fig. 7. This distribution basically conforms to a normal distribution with a mean of − 0.0221 and a standard deviation of 0.13601. This indicates that the rainfall events that induce the Bazhong landslides are discretely distributed above and below the fitted curve in the form of a log-normal distribution.

Figure 7 clearly shows that the logarithmic residuals are normally distributed. This indicates that it is reasonable to calculate the threshold under any subsequent non-exceedance probability using Eq. (5). On this basis, we performed threshold calculations under different non-exceedance probabilities, providing a data basis for subsequent fitting of the threshold surface.

### Continuous probability distribution function

Using the rainfall-induced landslide data from January 2011 to August 2019, the probability of a landslide occurrence caused by a rainfall event with continuous rainfall for more than 2 days can be determined using Eqs. (2) and (5). A three-dimensional surface fitting can then be performed using Eq. (6) to obtain the threshold surface of the rainfall-triggered landslide events under the continuous distribution probability for Bazhong City, as shown in Fig. 10a. The fitting results and the standard deviation of the fitting parameters are given in Table 2. The goodness of fit is 0.999, which is approximately equal to 1. This shows the superiority of using Eq. (6) for the probability distribution fitting and indicates that good fitting results are obtained.

**Table 2:** Bivariate polynomial parameter fitting results.

Parameter	Fitting results
$a$	4.846 ± 0.0530
$b$	0.087 ± 0.0027
$c$	− 0.047 ± 4.6853E-4

### Verification

**Figure 8:**
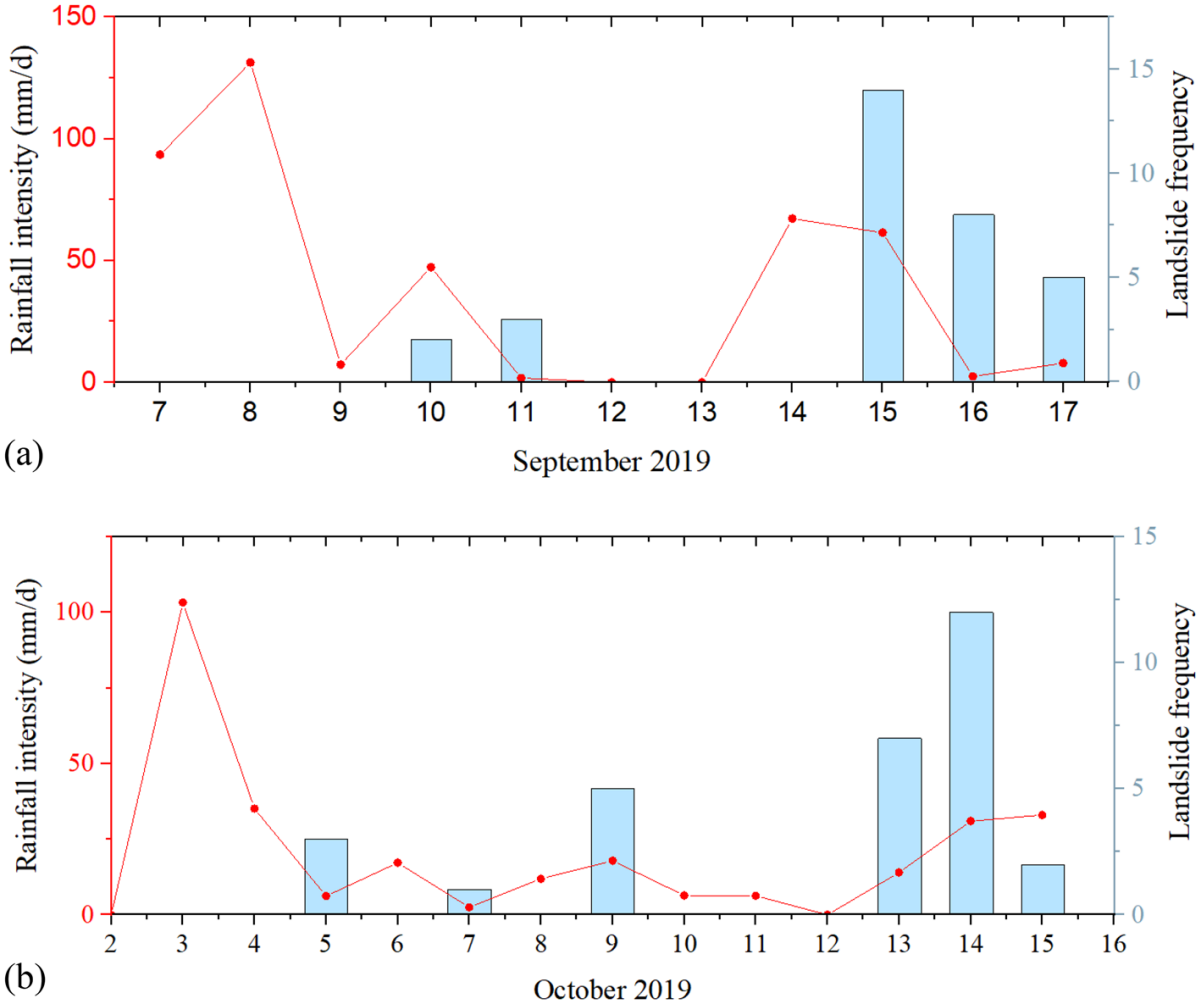
Two persistent rainfall-induced landslide events in September and October 2019.

In Bazhong City in 2019, following the peak landslide frequency caused by the flood in June and under the influence of the continuous rainfall conditions, landslide events occurred one after another at different times. In particular, during two rainfall events in September and October, the frequency of landslides again increased. Both rainfall events lasted for approximately 10 days. The two rainfall events together caused more than 60 landslides. See Fig. 8 for details.

**Figure 9:**
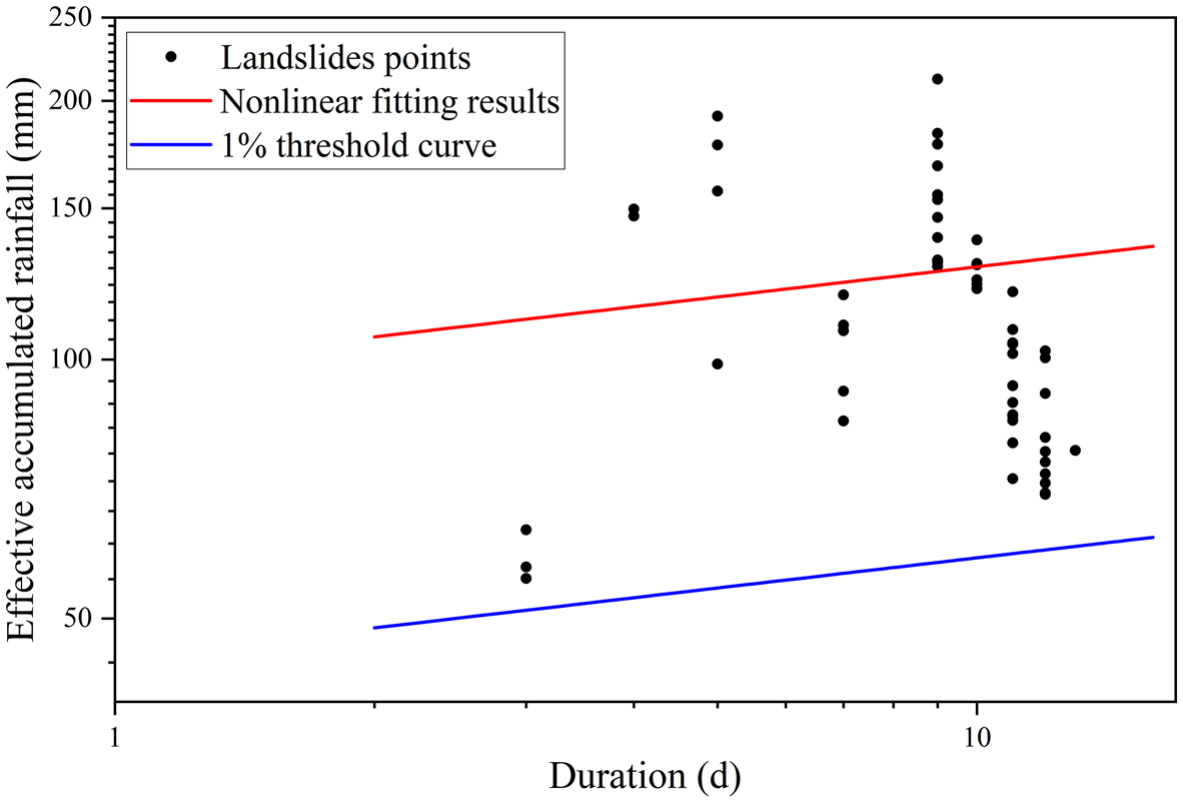
Rainfall threshold validation.

The threshold curve proposed in this study was verified using the rainfall-induced landslide data from September to October. As shown in Fig. 9, the rainfall events that induced landslides during these 2 months were discretely distributed above and below the nonlinear median fitting curve and the average logarithmic residual error of this part of the data was calculated to be − 0.07. In addition, this part of the data is above the 1% non-exceeding probability threshold curve. This illustrates the validity of the threshold curve obtained in this study.

**Figure 10:**
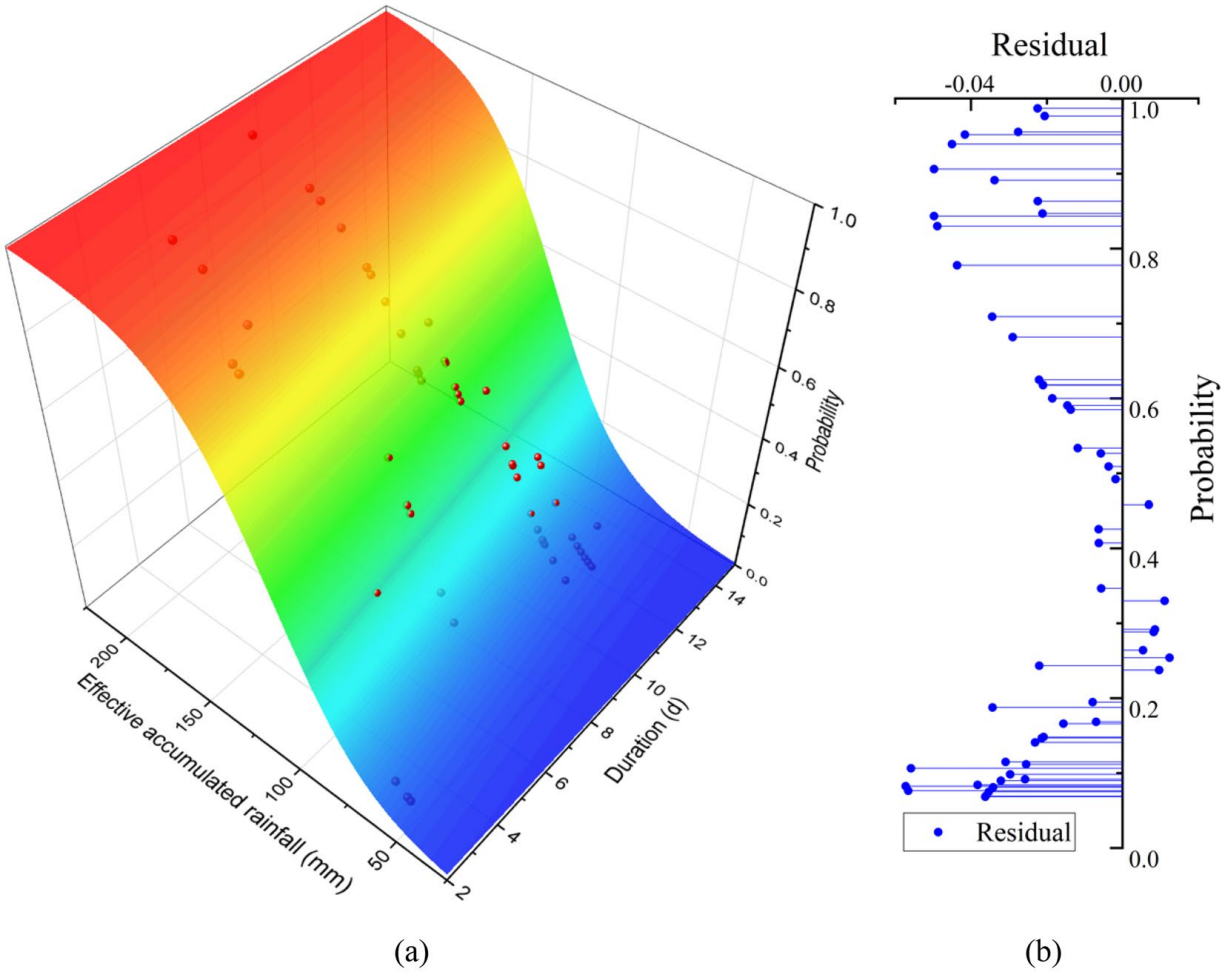
(**a**) Continuous probability distribution surface of rainfall-type landslides in Bazhong City and (**b**) residuals.

At the same time, according to the logarithmic residual value of this part of the data and the nonlinear fitting, the probability of each rainfall-induced landslide event in September and October 2019 was obtained, as shown in Fig. 10a. Compared with the fitting result of Eq. (6), the root mean square error is − 0.029, which shows the validity of the fitting result. In addition, Fig. 10b shows the landslide probability induced by this part of the rainfall event and the residual of the fitting surface. Interestingly, the residual change in the figure also shows an s-shape; however, there is fortunately no large residual value.

## Discussion

This paper describes a comprehensive method for establishing a continuous probability rainfall threshold based on the prediction interval principle. Several key issues concerning the workflow are discussed here.

### Impact of early rainfall

In this study, first, the relationships between the landslide events in Bazhong City and the rainfall intensity on the day of the event and the duration of the previous rainfall are analyzed. Table 1 and Fig. 5 clearly show that the rainfall inducing landslides in Bazhong City is mostly moderate rain lasting for 6–10 days, while light rain is very likely to occur on the day of a landslide. This emphasizes the important impact of early rainfall on landslide induction. Considering the discharge and evaporation processes of early rainfall, most studies focus on the effective accumulated rainfall. However, the selection of the effective rainfall coefficient in Eq. (1) is subjective. Additional scientific methods are needed to evaluate the rainfall infiltration process and obtain the appropriate effective rainfall coefficient. Especially in this study, when selecting the effective rainfall coefficient, considering that the constant value of relevant studies is 0.8^17,42–44^ and that the rainfall 6–10 days before a landslide in Bazhong City has a great impact, a greater value of 0.84 is used^38,45,46^. At present, this can be considered using complex decision-making algorithms^49^. At the same time, many studies have focused on the increase in soil moisture caused by early rainfall^50,51^. However, the simplest and most direct method is to establish a three-dimensional rainfall threshold model^52^. Through a simple and intuitive method, such a model can show the impact of early rainfall on landslide occurrence and can be based on threshold division, which has good feasibility and early warning performance.

### Uncertainty based on rainfall threshold warning

Figure 6 indicates that using a certain rainfall threshold curve with different non-exceeding probabilities has different prediction effects on whether rainfall events induce landslides. The adoption of a rainfall threshold with a low non-exceedance probability will lead to seriously inefficient identification of rainfall landslides. Moreover, most landslides cannot be effectively identified using a high non-exceedance probability rainfall threshold. However, in the verification of subsequent rainfall-triggered landslide events (Fig. 9), all landslide events occurred on the 1% non-exceeding probability threshold curve, while only one-third of landslides occurred on the 50% non-exceeding probability threshold. This makes it difficult to choose which of the two is more suitable as the rainfall threshold for landslides in Bazhong City. Because it considers the influences of many possible factors, such as vegetation conditions, slope, elevation, and weathering degree, the rainfall threshold has great uncertainty. This problem has also been noted in previous studies^21,24^, but further research has not yielded a threshold function of the continuous distribution probability. Given a certain rainfall condition, the probability of landslide occurrence can be directly obtained, which is more suitable for project decision-making.

Comparing Figs. 6 and 9, the selection of a rainfall threshold for inducing landslides should clearly be made based on an extensive collection of local rainfall events; this should not only prevent high false alarm rates but also improve the identification of landslide events. Some relatively good related research work has been carried out; for example, Segoni et al. developed the MaCumBA software to obtain the rainfall threshold with the smallest false alarm rate through multiple operations and reverse analyses^53^. Such methods should be an indispensable component of future rainfall threshold research.

### Further study

There are still some deficiencies in the process of carrying out the above research, and future engineering early warning research could be carried out based on the following.

1. The accuracy of analyzing the probability of rainfall-induced landslides in a certain area using historical empirical data depends largely on the collection of sample data. Compared with other studies with thousands of landslide records, this study warrants further data collection and records. Only by constantly expanding the sample data and updating the probability threshold of rainfall landslides can geological disaster early warning work be improved.
2. To obtain a more scientific rainfall threshold, we need to consider a large number of rainfall events, including those that triggered landslides and those that did not. On the basis of this, landslide events can be effectively identified to minimize the rate of misjudgment. Therefore, further study is necessary to obtain the lowest false alarm rate based on continuous probability threshold analyses.
3. In the same area, even though there are similar stratigraphic conditions, conditions such as the slope, elevation, and vegetation cover differ, which will lead to uncertainties in the rainfall threshold. Risk analyses of certain areas should be performed by obtaining continuous probability rainfall thresholds combined with landslide susceptibility analyses. At the same time, the landslide warning effect can gradually be improved by combining real-time or climate prediction technologies.

## Conclusions

On the basis of the collection of landslide hazards and ERA5-Land high-resolution rainfall data in Bazhong City from 2011 to 2019, this study used the concept of effective cumulative rainfall to conduct a refined rainfall threshold analysis of landslide hazards in Bazhong City. The main conclusions are as follows.

1. Landslide hazards in Bazhong City primarily occur in the period of June–October when the monthly average rainfall is high; events are especially concentrated in the period of June–July. Moderate rain-induced landslide hazards lasting approximately 10 days are the main type of event, accounting for approximately 70% of the collected data.
2. A power function fitting of the rainfall and landslide hazard events in Bazhong City was performed via nonlinear regression, and a threshold curve of ${E}_{\mathrm{L}}=98.05{\mathrm{D}}^{0.1166}$ was obtained. The threshold curve under the 1% non-exceeding probability is ${E}_{L-0.01}=44.97{\mathrm{D}}^{0.1166}$. The threshold results were verified against two sustained rainfall events in September and October 2019, and the rainfall events were all above the 1% threshold line.
3. This study proposes using the continuous probability rainfall threshold surface for quantitative analyses. On the basis of the Sigmoid function, a continuous probability distribution function of rainfall-induced landslides was established and good fitting results were obtained. Using this method, we can preliminarily solve the problem of selecting an appropriate non-exceeding probability rainfall threshold.

The above research results provide a powerful tool to aid in the early warning of rainfall-induced landslides in Bazhong City. As a typical regional case prone to rainfall-induced landslides, the study of Bazhong City can provide a reference for studies of rainfall-induced landslides in other regions.

## Data Availability

The data that support the findings of this study are available from the corresponding author upon request. We used publicly available terrain data (https://earthexplorer.usgs.gov/) and rainfall data from the European Centre for Medium-Range Weather Forecasts (https://www.ecmwf.int/en/era5-land).
